# Alternative promoter usage and mRNA splicing pathways for parathyroid hormone-related protein in normal tissues and tumours.

**DOI:** 10.1038/bjc.1995.397

**Published:** 1995-09

**Authors:** J. Southby, L. M. O'Keeffe, T. J. Martin, M. T. Gillespie

**Affiliations:** St. Vincent's Institute of Medical Research, St. Vincent's Hospital, Victoria, Australia.

## Abstract

**Images:**


British Journal of Cancer (1995) 72, 702-707

© 1995 Stockton Press All rights reserved 0007-0920/95 $12.00

### Alternative promoter usage and mRNA splicing pathways for parathyroid hormone-related protein in normal tissues and tumours

J Southby, LM O'Keeffe, TJ Martin and MT Gillespie

*St. Vincent's Institute of Medical Research and The University of Melbourne Department of Medicine, St. Vincent's Hospital, 41 Victoria Parade, Fitzroy 3065, Victoria, Australia.*

**Summary** The parathyroid hormone-related protein (PTHrP) gene consists of nine exons and allows the production of multiple PTHrP mRNA species via the use of three promoters and 5' and 3' alternative splicing; as a result of 3' alternative splicing one of three protein isoforms may be produced. This organisation has potential for tissue-specific splicing patterns. We examined PTHrP mRNA expression and splicing patterns in a series of tumours and normal tissues, using the sensitive reverse transcription-polymerase chain reaction (RT-PCR) technique. Use of promoter 3 and mRNA specifying the 141 amino acid PTHrP isoform were detected in all samples. Transcripts encoding the 139 amino acid isoform were detected in all but two samples. Use of promoters 1 and 2 was less widespread as was detection of mRNA encoding the 173 amino acid isoform. While different PTHrP splicing patterns were observed between tumours, no tissue- or tumour-specific transcripts were detected. In comparing normal and tumour tissue from the same patient, an increase in the number of promoters utilised was observed in the tumour tissue. Furthermore, mRNA for the PTH/PTHrP receptor was detected in all samples, thus the PTHrP produced by these tumours may potentially act in an autocrine or paracrine fashion.

**Keywords**: mRNA splicing; breast cancer; renal cancer; lung cancer; parathyroid hormone-related protein

Parathyroid hormone-related protein (PTHrP) was initially discovered as a tumour product involved in malignancy-associated hypercalcaemia and has since been identified in a wide range of tumours as well as in normal tissues (Martin *et al*., 1991; Moseley and Gillespie, 1992). The protein exhibits sufficient N-terminal homology with PTH to interact with the PTH receptor, accounting for its PTH-like actions in promoting hypercalcaemia (Abou-Samra *et al*., 1992). The human PTH/PTHrP receptor has been cloned from kidney and osteoblast-like osteosarcoma cell cDNA libraries and was found to be a member of a family of seven transmembrane domain, G-protein-linked receptors (Schipani *et al*., 1993). The remainder of the PTHrP sequence diverges from that of PTH and possesses non-PTH-like actions, including roles in placental calcium transport (Rodda *et al*., 1988), inhibition of osteoclastic bone resorption *in vitro* (Fenton *et al*., 1991) and cell growth and/or differentiation (Henderson *et al*., 1991).

The human *PTHrP* gene consists of nine exons, of which only two are present in all PTHrP transcripts: exon V, encoding the prepro region of the protein, and exon VI, which encodes the majority of the coding region for the mature protein up to residue 139, where a splice donor site is located. Readthrough of exon VI to exon VII introduces a stop codon and produces mRNA for the 139 amino acid isoform, while splicing of exon VI to exon VIII or IX results in mRNA specifying the 173 and 141 amino acid isoforms respectively (Suva *et al*., 1987; Mangin *et al*., 1988*a*,*b*, 1989; Thiede *et al*., 1988; Yasuda *et al*., 1989). Thus, alternative 3' splicing allows the production of mRNA specifying three isofoms of PTHrP which differ in their C-terminal regions. Three promoters have been identified in the gene: two TATA promoters, 5' to exons I and IV (P1 and P3 respectively) (Thiede *et al*., 1988; Mangin *et al*., 1989, 1990; Suva *et al*., 1989; Yasuda *et al*., 1989), and a GC-rich promoter, 5' to exon III (P2) (Vasavada *et al*., 1993). In addition, exon II may be included in or excluded from transcripts originating from promoter 1 (Mangin *et al*., 1990; Glatz *et al*., 1994), or exon I may be spliced directly to exon V (Glatz *et al*., 1994). We and others have demonstrated in cell lines that more than one promoter may be utilised and alternative splicing events occur (Mangin *et al*., 1990; Southby *et al*., 1993; Vasavada *et al*., 1993; Brandt *et al*., 1994; Glatz *et al*., 1994). The presence of three promoters and 5' and 3' alternative splicing suggests that PTHrP expression may be differentially regulated by growth factors during development or in a tissue-specific manner. It has been suggested that tissue-specific splicing of PTHrP mRNA does occur (Mangin *et al*., 1989; Campos *et al*., 1992; Brandt *et al*., 1994), however these studies utilised only cell lines (Brandt *et al*., 1994) or a small number of primary tumours (Mangin *et al*., 1989; Campos *et al*., 1992). To investigate whether PTHrP mRNA is alternatively spliced in a tissue-specific manner we have examined the PTHrP mRNA splicing patterns in a series of tumours and normal tissues, using the reverse transcription-polymerase chain reaction (RT-PCR) technique with PTHrP exon-specific primers. We found that while the tumours exhibited different splicing patterns, these differences were not tumour type-specific. Since PTHrP may have local actions, as well as its hypercalcaemic actions observed in malignancy-associated hypercalcaemia, we also examined the tissue and tumour samples for mRNA encoding the PTH/PTHrP receptor.

### Materials and methods

### *Tumour samples*

Tumours, and normal tissue where available, were collected at biopsy or surgery from patients attending the Heidelberg Repatriation Hospital or St. Vincent's Hospital, in the normal course of their management. The tissues were immediately placed on dry ice and stored at − 70°C. The tumour samples included 13 breast tumours [all infiltrating ductal carcinomas, with three tumours also containing a ductal carcinoma *in situ* component (B1, B4, B11)], five renal cortical carcinomas (R1-R5) and one renal carcinoma metastasis to lung (R6), 13 lung tumours [eight squamous cell carcinomas (L1-L8), two large cell carcinomas (L9, L10), one small cell carcinoma (L11) and two adenomas (L12, L13)], one oesophageal squamous cell carcinoma (E), one hepatoma (H) and five parathyroid samples [one hyperplasia (PT1) and four adenomas (PT2-PT5)]. Normal tissue included two renal and four lung samples adjacent to the tumour sample and one thyroid sample (T) which was

Correspondence: MT Gillespie
Received 19 January 1995; accepted 28 April 1995.

obtained in the course of removal of the parathyroid gland. The calcium status (normo-, hypo- or hypercalcaemic) of the patients from which biopsies were obtained is not known.

*RNA extraction, cDNA synthesis and polymerase chain reaction (PCR)*

Total RNA was extracted from the tissues by the guanidinium thiocyanate-phenol-chloroform method (Chomczynski and Sacchi, 1987). An aliquot of 5 μg of total RNA was reverse transcribed from an oligo (dT) primer using 0.5 μg of oligo (dT) primer (Promega, WI, USA), 15 units of avian myeloblastosis virus reverse transcriptase (Promega), 5 μl of 5 × reverse transcription buffer, 0.25 mM each dATP, dCTP, dGTP and dTTP (Pharmacia, Uppsala, Sweden), 10 mM dithiothreitol and sterile distilled water to a total volume of 25 μl.

One twenty-fifth of the reverse transcription (RT) reaction was used per PCR in a 20 μl reaction mixture containing 50 pmol of each primer, 25 μM each dATP, dGTP, dCTP and dTTP, 2 μl of 10 × reaction buffer, 1 unit of *Taq* DNA polymerase (Boehringer Mannheim, Mannheim, Germany) and sterile distilled water and overlaid with 50 μl of paraffin oil. Amplification was performed in a Perkin Elmer DNA thermal cycler 480, with 40 cycles of denaturation at 95°C for 30 s, annealing at 55°C for 30 s and extension at 72°C for 1 min. PCR products were resolved on a 2% (w/v) agarose gel, transferred to Hybond-N membrane (Amersham, Buckinghamshire, UK) and hybridised with a $^{32}P$-labelled (Amersham) oligonucleotide probe in order to confirm the specificity of the reaction. All negative control reactions (e.g. primers without DNA, PCR on RNA which had not been reverse transcribed) were negative by gel and Southern hybridisation.

Oligonucleotides utilised for PCR and authentication of PCR products were synthesised on an Applied Biosystems (CA, USA) DNA synthesiser model 381A. PTHrP oligonucleotides were: obrf 15.8 [5′-TGAAGGAAGAATCGTCGCC-GTAAATCTTGGATGGAC-3′ antisense strand-specific to exon VI, nucleotides +147 to +182 from the start of translation with respect to cDNA sequence (Suva *et al.*, 1987)]; obrf 15.83 [5′-GTTGGAGTAGCCGGTTGCTA-3′, sense strand-specific to exon IV, nucleotides −211 to −192 from the start of translation with respect to genomic sequence (Suva *et al.*, 1989)]; obrf 15.84 [5′-CGGTGTTCCTGCTGA-GCTA-3′ sense strand-specific to exon V, nucleotides +35 to +53 from the start of translation with respect to cDNA sequence (Suva *et al.*, 1987)]; obrf 15.89 [5′-TGCGATCAG-ATGGTGAAGGA-3′, antisense strand-specific to exon VI, nucleotides +176 to +195 from the start of translation with respect to cDNA sequence (Suva *et al.*, 1987)]; obrf 15.90 [5′-CACAATCGATAGAGATAC-3′, antisense strand-specific to exon VII, nucleotides +620 to +637 from the start of translation with respect to cDNA sequence (Yasuda *et al.*, 1989)]; obrf 15.91 [5′-GAGATCATTAGTTGCATATG-3′, antisense strand-specific to exon VIII, nucleotides +580 to +599 from the start of translation with respect to cDNA sequence (Yasuda *et al.*, 1989)]; obrf 15.92 [5′-CAGAATCC-TGCAATATGTCC-3′, antisense strand-specific to exon IX, nucleotides +558 to +577 from the start of translation with respect to cDNA sequence (Yasuda *et al.*, 1989)]; obrf 15.93 [5′-AGGTACCTGCTTTCTAATA-3′, sense strand-specific to exon I, nucleotides −3210 to −3191 from the start of translation with respect to genomic sequence (Mangin *et al.*, 1990)]; obrf 15.95 [5′-TTCTCCGGCAGGTTTG-3′, sense strand-specific for promoter 2 transcripts, nucleotides −783 to −768 from the start of translation with respect to genomic sequence (Vasavada *et al.*, 1993)]. Oligonucleotides specific for the human PTH/PTHrP receptor were obrf 15.112 [5′-CCTCTTTGGCGTCCACTACATTG-3′, sense strand-specific, nucleotides +1273 to +1295 (Schipani *et al.*, 1993)]; obrf 15.113 [5′-TTGAGGAACCCATCGTCCTTG-3′, antisense strand-specific, nucleotides + 1702 to + 1722 (Schipani *et al.*, 1993)] and the detection oligonucleotide obrf 15.114 [5′-GGCAAGTCCAGATGCACTATGAG-3′, sense strand-specific, nucleotides +1337 to +1360 (Schipani *et al.*, 1993)]. Oligonucleotides used to amplify a 414 bp fragment of glyceraldehyde-3-phosphate dehydrogenase (GAPDH) were: GAPDH-4 [5′-CATGGAGAAGGCTGGGGCTC-3′, nucleotides 306–325 (Tso *et al.*, 1985)] and GAPDH-3 [5′-CACTGACACGTTGGCAGTGG-3′, nucleotides 701–720 (Tso *et al.*, 1985)] and products were verifed with an internal sense strand oligonucleotide, GAPDH-1 [5′-GCTGTGGG-CAAGGTCATCCC-3′, nucleotides 640–659 (Tso *et al.*, 1985)]. Oligonucleotides for GAPDH amplification were designed to different exons [GAPDH-4 across exons 5 and 6 and GAPDH-3 to exon 8 (Ercolani *et al.*, 1988)], allowing for the detection of GAPDH mRNA only.

### Results

The sensitive RT–PCR method was used to determine PTHrP promoter usage and mRNA splicing patterns in a series of tumour and normal tissues. Figure 1 shows the organisation of the human *PTHrP* gene, its splicing pathways and the exon-specific primers used for PCR. Total RNA was extracted from 39 tumours and seven samples of normal tissue and subjected to RT–PCR with the PTHrP primers and primers specific for the PTH/PTHrP receptor. In addition, RT–PCR with GAPDH primers was performed as a control for the quality of the extracted RNA and for the reverse transcription reaction. Since the aim of this study was purely qualitative, i.e. to determine which splicing patterns were utilised, PCR was carried out under saturating, non-quantitative, conditions with the PTHrP- and PTH/PTHrP receptor-specific primers. The majority of PCR products were clearly visible after 30 cycles of PCR, however, in order to ensure detection of all the splicing patterns utilised, the PCRs were conducted at 40 cycles, which had been determined previously to be saturating for the PTHrP primers. Amplification of GAPDH cDNA was carried out at 30 cycles which was found to be saturating for the GAPDH primers.

PCR was initially performed using primers to the two invariant exons of PTHrP mRNA, exons V and VI (Figure 1), in order to assess PTHrP mRNA production (Figure 2). A band of the appropriate size (161 bp) was observed for each sample, which hybridised with an internal PTHrP oligonucleotide (obrf 15.8), confirming that the PCR products were the results of specific amplification of PTHrP mRNA. Thus all of the tumour and normal tissues examined expressed PTHrP mRNA (Figure 2).

To address which of the PTHrP isoforms the tumours were capable of producing (i.e. 139, 141 or 173 amino acid isoforms), the 3′ alternative splicing patterns were investigated by amplifying between the common exon V and the alternative exons VII, VIII and IX (Figure 1). All of the breast tumours studied used each of the three 3′ splicing pathways (Figure 2a, B1–B13) and thus expressed mRNA specifying each of the three PTHrP isoforms. A similar pattern of 3′ splicing was observed with the renal samples (Figure 2b). Splicing of exon VI to each of the three alternative 3′ exons occurred in both the normal renal tissue (N1 and N2) and the tumour samples (R1–R6). Three of the normal lung samples (Figure 2c, N1–N3) and the 13 lung tumours (L1–L13) utilised the exon VII and exon IX splicing pathways. The fourth normal lung sample (N4) used the exon IX but not the exon VII splicing pathway. Exon VI was spliced to exon VIII in only three of the normal samples (N1, N3, N4) and nine of the tumours (Figure 2c, L1, L2, L3, L5, L6, L9, L10, L12 and L13). The three splicing pathways were utilised in the oesophageal squamous cell carcinoma (E), the hepatoma (H) and three of the five parathyroid samples (PT1–PT3), but exon VIII-containing mRNA was not detected in the normal thyroid tissue (T) nor in two of the five parathyroid samples (PT4 and PT5; Figure 2d). Thus, all tissues examined contained mRNA specifying the 141 amino acid isoform of PTHrP and all but two samples (lung N4 and PT5) contained mRNA encoding the 139 amino acid isoform, while the utilisation of the exon VIII splicing pat-

| PCR primer combination | Transcripts detected | Exons | Size of product (bp) |
|---|---|---|---|
| obrf 15.84–obrf 15.89 | All transcripts | V–VI | 161 |
| obrf 15.84–obrf 15.90 | 139 aa isoform | V–VI–VII | 603 |
| obrf 15.84–obrf 15.91 | 173 aa isoform | V–VI–VIII | 565 |
| obrf 15.84–obrf 15.92 | 141 aa isoform | V–VI–IX | 543 |
| obrf 15.93–obrf 15.89 | P1 transcripts | I–II–III–V–VI | 739 |
| | | I–III–V–VI | 646 |
| | | I–V–VI | 406 |
| obrf 15.95–obrf 15.89 | P2 transcripts | III–V–VI | 468 |
| obrf 15.83–obrf 15.89 | P3 transcripts | IV–V–VI | 242 |

**Figure 1** Diagrammatic representation of the genomic organisation of the *PTHrP* gene. The coding regions and the untranslated sequences are indicated by the closed and open boxes respectively. Indicated above the map are the three promoters. P1 (5′ to exon I) and P3 (5′ to exon IV) are TATA promoters and P2 (5′ to exon III) is a GC-rich promoter. Potential splicing events are shown. The arrows below the map indicate the orientation and location of the primers used for RT–PCR analysis of PTHrP transcripts and the antisense oligonucleotide used to detect PTHrP products (obrf 15.8) is represented below exon VI. Also shown are the PCR primer combinations used for Figure 2, the transcripts they detect along with the predicted isoform produced, the exonic sequences contained in the amplified product and the size of the PCR product in bp.

**Figure 2** PCR analysis of PTHrP mRNA splicing patterns and for the detection of PTH/PTHrP receptor mRNA. PCR amplification of PTHrP cDNA was performed between the exons indicated, using the primers shown in Figure 1. PCR amplification of PTH/PTHrP receptor cDNA was carried out using primers obrf 15.112 and obrf 15.113 and amplification of GAPDH cDNA with primers GAPDH-3 and GAPDH-4. PCR products were resolved on a 2% (w/v) agarose gel, transferred to a nylon membrane and hybridised with an internal oligonucleotide; obrf 15.8 (PTHrP), obrf 15.114 (PTH/PTHrP receptor) or GAPDH-1. PCR analysis of PTHrP mRNA splicing patterns and PTH/PTHrP receptor mRNA was conducted on (**a**) 13 breast tumours (B1–B13), (**b**) two normal renal samples (N1 and N2) and six renal tumours (R1–R6), (**c**) four normal lung samples (N1–N4) and 13 lung tumours (L1–L13), (**d**) an oesophageal squamous cell carcinoma (E), hepatoma (H), normal thyroid (T), one parathyroid hyperplasia and four parathyroid adenomas (PT1–PT5). The autoradiographs are at different exposure times (2–16 h) in order to optimise the visibility of the bands of weaker intensity and the reactions were performed twice with similar results.

tern and hence the production of the 173 isoform was more variable.

To differentiate between the use of the PTHrP promoters, PCR was carried out using primers to the alternative 5′ exons, specific to transcripts derived from each of the promoters (P1, P2 and P3), and the common exon VI (Figure 1). PCR amplification between the primers specific for exon I (obrf 15.93) and exon VI (obrf 15.89) detects promoter 1-specific transcripts and may result in amplification of three different products which indicate alternative splicing of exons II and III in the primary transcript. The three products represent (a) splicing of exons I, II, III, V and VI (739 bp), (b) splicing of exons I, III, V and VI (with the exclusion of exon II; 646 bp) and (c) splicing of exon I directly to exons V and VI (406 bp) (Glatz *et al.*, 1994). We did not detect in any of the normal tissues or tumours any products equivalent to the 406 bp fragment resulting from splicing of exons I–V. Exon IV-containing transcripts are the result exclusively of P3 activity, thus exon I- and IV-containing transcripts are exquisite markers for P1 and P3 activity respectively. P2 transcripts are initiated 11 nucleotides 5′ to the splice acceptor site of exon III (Vasavada *et al.*, 1993). For detection of P2 activity, the 5′ primer was synthesised to this region in order to distinguish between P2 transcripts and those containing exon III as a result of initiation from P1 and subsequent splicing to exon III (Glatz *et al.*, 1994). Thus amplification between exons I, III and IV and exon VI allowed the usage of P1, P2 and P3 respectively, to be determined (Glatz *et al.*, 1994).

Promoter 1 transcripts were detected in seven of the 13 breast tumours, as determined by amplification between exons I and VI (Figure 2a). Two products were identified in four of these tumours (B1, B4, B5 and B8), the larger product (739 bp) corresponding to P1 transcripts containing exon II and the smaller (646 bp) representing P1 transcripts in which exon II has been spliced out; the products are not resolved on this gel. The other three tumours (Figure 2a, B7, B10 and B12) in which P1 was active appeared to use only the I–II–III–V–VI splicing pathway. One of the two normal renal samples (N1) and all of the six renal tumours (R1–R6) contained P1 transcripts (Figure 2b), with the normal tissue and three of the tumours (R3–R5) utilising the I–II–III–V–VI splicing pathway only. Two renal tumours (R1 and R6) appeared to produce P1 transcripts both with and without exon II and the remaining tumour (R2) produced only P1 transcripts in which exon II had been spliced out. P1 transcripts were present in one sample of normal lung tissue (N1) and in six of the lung tumours (Figure 2c). P1 transcripts both with and without exon II were identified in the normal tissue (N1) and three of the lung tumours (L5, L6 and L9), while P1 only transcripts containing exon II were detected in the other three lung tumours (L1, L2 and L11). P1 transcripts containing exon II were identified in the oesophageal tumour (E), the hepatoma (H) and one of the parathyroid samples (PT1; Figure 2d). The other parathyroid sample in which P1 was active (PT3) contained P1 transcripts both with and without exon II (Figure 2d).

PCR was performed between exons III and VI to determine usage of the GC-rich promoter. Promoter 2-specific transcripts were detected in eight of the 13 breast tumours (Figure 2a) and all of the renal samples except one tumour sample (Figure 2b, R4). P2-initiated transcripts were not detected in the normal lung tissues but were present in five of the lung tumours (L3, L5, L6, L9 and L11); four of the lung tumours (L5, L6, L9 and L11) contained both P1- and P2-initiated transcripts (Figure 2c). The oesophageal tumour (E) and two of the five parathyroid samples (PT2, PT3) also contained P2 transcripts (Figure 2d).

In contrast to the more restricted utilisation of P1 and P2, P3-initiated transcripts were detected in all of the tissue samples. While many of the samples utilised all three promoters, there were examples of use of P1 but not P2 and vice versa.

Overall, the use of P3 and splicing to exon IX appeared to be common to all normal and tumour tissues and all except two samples used the exon VII splicing pathway but there was differential use of the alternative exon VIII and of the two upstream promoters P1 and P2. Each group of tumours exhibited an array of splicing patterns and there did not appear to be a splicing preference particular to one tissue or tumour type.

For the two normal kidney samples and three of the four normal lung samples there was also tumour tissue from the same patient (renal: N1 R2, N2 R3; lung: N1 L9, N2 L2, N4 L6). The same splicing patterns were observed in the normal and tumorous renal tissues except for the use of P1 in one pair: P1 was not active in one normal renal sample (N2) but P1 transcripts were detected in the tumour counterpart (R3; Figure 2b). In comparing the normal and tumour lung samples (Figure 2c), two normal lung samples used P1 and P3 (N1) or P3 only (N4) while their tumour counterparts utilised all three promoters (L9 and L6 respectively). Promoter 3 only was active in the third normal lung sample (N2) while P1 and P3 were active in the tumour (L2). One normal sample (N4) lacked exon VII-containing mRNA and another (N2) lacked exon VIII-containing mRNA while the corresponding tumours (L6 and L2) utilised all three 3′ splicing pathways. This would result in the two lung tumour samples, L2 and L6, producing each of the three PTHrP protein isoforms, while their corresponding normal samples, N2 and N4, are predicted to only produce the 139 and 141 or the 173 and 141 amino acid isoforms respectively.

In addition, the tissue and tumour samples were examined for the presence of PTH/PTHrP receptor mRNA. Amplification with two receptor-specific primers detected mRNA for the PTH/PTHrP receptor in all of the samples (Figure 2).

RT–PCR with GAPDH primers was performed as a control for the quality of the extracted RNA and for the reverse transcription reaction. Each of the tissue and tumour samples displayed a band on PCR with the GAPDH primers which hybridised with the internal GAPDH oligonucleotide, GAPDH-1 (Figure 2).

### Discussion

The human *PTHrP* gene has a complex organisation, with nine exons, three promoters and multiple splicing pathways (Martin *et al.*, 1991; Moseley and Gillespie, 1992). Initial studies have raised the possibility of tissue-specific splicing of PTHrP transcripts (Mangin *et al.*, 1989; Brandt *et al.*, 1992, 1994; Campos *et al.*, 1992), however these studies were limited by the number of samples analysed or failure to examine the use of all three promoters at a time when the complete exonic structure had not been resolved; the GC-rich promoter, P2, had not been identified. Mangin *et al.* (1989) assessed the PTHrP mRNA splicing pathways of four human tumours of different types and found that the tumours displayed different 3′ splicing pathways, leading to the suggestion that the PTHrP mRNA splicing patterns were tumour-specific. Campos *et al.* (1992) examined the use of the two TATA promoters (P1 and P3) in 17 neoplastic and 11 non-neoplastic tissues, finding that most of the samples which expressed PTHrP utilised both P1 and P3. However utilisation of the GC-rich promoter was not addressed. Vasavada *et al.* (1993) in examining the relative use of the three promoters by a number of cell lines and tissues found P2 and P3 to be widely utilised with use of P1 more restricted. Brandt *et al.* (1994) investigated both *PTHrP* promoter usage and 3′ splicing pathways of seven cell lines and found preferential use of P2 and the splicing pathway generating the 139 amino acid isoform and suggested that use of P1 and P3 and the 173 and 141 amino acid isoform splicing pathways may be regulated in a cell line-regulated manner. A similar use of P2 and the 139 amino acid isoform splicing pathway was observed in normal human amnion (Brandt *et al.*, 1992). We have undertaken a more extensive study using RT–PCR with exon-specific primers to cover every promoter and 3′ splicing choice and obtained multiple examples of each tumour type. We and others have previously identified PTHrP mRNA or protein in human breast, renal, lung, oesophageal and liver tumours, normal thyroid and kidney and in parathyroid hyperplasia and adenoma by Northern blot analysis, RT-PCR, or immunohistochemistry (Mangin *et al.*, 1988*a*, 1989; Ikeda *et al.*, 1988; Danks *et al.*, 1989, 1990; Southby *et al.*, 1990; Campos *et al.*, 1992). Immunohistochemical staining for PTHrP in breast, renal and lung tumours was mostly confined to the tumour cells, with only occasional staining observed in the stroma or in blood vessels (Danks *et al.*, 1989, 1990; Southby *et al.*, 1990), thus indicating that the PTHrP mRNA detected in this study is most likely to be tumour derived. We found that each group of tumours exhibited an array of different splicing patterns. Each of the samples examined contained PTHrP mRNA. Alternative 3′ splicing results in the production of PTHrP isoforms of 139, 173 and 141 amino acids, depending on whether exon VII, VIII or IX, respectively, is incorporated as the 3′ exon. Splicing of exon VI to exon IX was evident in all tissue samples and splicing of exon VI to exon VII occurred in all but two samples, suggesting that the 141 and 139 amino acid isoforms are common. The exon VIII splicing pathway, and hence the 173 amino acid isoform, was less often present. *PTHrP* gene transcription may be initiated from three promoters: P1, the upstream TATA promoter, P2, the GC-rich promoter 5′ to exon III and P3, the downstream TATA promoter. Each of the tissue and tumour samples contained P3-initiated transcripts, indicating widespread use of P3, but the use of P1 and P2 was more limited. Hence, while all samples used P3 and exon IX, there was differential, however not tissue-specific, use of P1 and P2 and the alternative exon VIII. This data contradicts that of Brandt *et al.* (1994) who suggested that the GC-rich promoter, P2, is the most active promoter. These studies were performed on RNA extracted from only seven cell lines and it may be that results from cell lines cannot readily be extrapolated to primary tumours. Mangin *et al.* (1989) observed different 3′ splicing patterns amongst four different tumour types; however only one sample of each type was studied. In studying a larger number of tumours we found that different splicing patterns were utilised within a group of tumours of the same type and could not demonstrate tissue- or tumour-specific splicing patterns and, by inference, we cannot predict that there are tissue- or tumour-specific isoforms of PTHrP.

In a number of cases, normal and tumour tissue were obtained from the same patient. In each case where the splicing patterns differed, there was an increase in the number of promoters used and, in two cases, there was also an increase in the number of 3′ splicing pathways utilised in some lung tumour samples, compared with the normal tissue. This is perhaps a result of altered regulation as a consequence of transformation to the cancer state and suggests a general increase in *PTHrP* transcription in the tumour cells which possibly contributes to the overexpression of PTHrP in those tumours which cause hypercalcaemia.

Identification of PTH/PTHrP receptor mRNA in the normal kidney and renal tumours was expected, given that the kidney is one of the targets of PTH action. Breast and lung are not classical sites of PTH action, however PTH/PTHrP receptor mRNA has been detected in many rat tissues, including breast and lung (Urena *et al.*, 1993). Since PTHrP has been detected in vascular smooth muscle (de Papp and Stewart, 1993) and shown to induce vascular relaxation (Winquist *et al.*, 1987) it is possible that the PTH/PTHrP receptor mRNA detected is derived from the blood vessels of the tissues. However if the PTH/PTHrP receptor is present in the same tissues which produce PTHrP it suggests that if the PTHrP is secreted it may potentially act on the same cell or neighbouring cells, possibly affecting the growth and differentiation of the tumour.

**Acknowledgements**
This work was supported by the National Health and Medical Research Council of Australia. JS was a recipient of an Australian Postgraduate Research Award and MTG was a recipient of a RD Wright Research Fellowship of the NH and MRC.

**References**

ABOU-SAMRA A-B, JUPPNER H, FORCE T, FREEMAN MW, KONG X-F, SCHIPANI E, URENA P, RICHARDS J, BONVENTRE JV, POTTS Jr JT, KRONENBERG HM AND SEGRE GV. (1992). Expression cloning of a common receptor for parathyroid hormone and parathyroid hormone-related peptide from rat osteoblast-like cells: a single receptor stimulates intracellular accumulation of both cAMP and inositol triphosphates and increases intracellular free calcium. *Proc. Natl Acad. Sci. USA*, **89**, 2732-2736.

BRANDT DW, BRUNS ME, BRUNS DE, FERGUSON II JE, BURTON DW AND DEFTOS LJ. (1992). The parathyroid hormone-related protein (PTHrP) gene preferentially utilizes a GC-rich promoter and the PTHrP 1-139 coding pathway in normal human amnion. *Biochem. Biophys. Res. Comm.*, **189**, 938-943.

BRANDT DW, WACHSMAN W AND DEFTOS LJ. (1994). Parathyroid hormone-like protein: alternative messenger RNA splicing pathways in human cancer cell lines. *Cancer Res.*, **54**, 850-853.

CAMPOS RV, WANG C AND DRUCKER DJ. (1992). Regulation of parathyroid hormone-related peptide (PTHrP) gene transcription: cell- and tissue-specific promoter utilization mediated by multiple positive and negative *cis*-acting DNA elements. *Mol. Endocrinol.*, **6**, 1642-1652.

CHOMCZYNSKI P AND SACCHI N. (1987). Single-step method of RNA isolation by acid guanidinium thiocyanate-phenol-chloroform extraction. *Anal. Biochem.*, **162**, 156-159.

DANKS JA, EBELING PR, HAYMAN J, CHOU ST, MOSELEY JM, DUNLOP J, KEMP BE AND MARTIN TJ. (1989). Parathyroid hormone-related protein: immunohistochemical localization in cancers and in normal skin. *J. Bone Min. Res.*, **4**, 273-278.

DANKS JA, EBELING PR, HAYMAN JA, DIEFENBACH-JAGGER H, COLLIER FMCL, GRILL V, SOUTHBY J, MOSELEY JM, CHOU ST AND MARTIN TJ. (1990). Immunohistochemical localization of parathyroid hormone-related protein in parathyroid adenoma and hyperplasia. *J. Pathol.*, **161**, 27-33.

DE PAPP AE AND STEWART AF. (1993). Parathyroid hormone-related protein. A peptide of diverse physiologic functions. *Trends Endocrinol. Metab.*, **4**, 181-187.

ERCOLANI L, FLORENCE B, DENARO M AND ALEXANDER M. (1988). Isolation and complete sequence of a functional human glyceraldehyde-3-phosphate dehydrogenase gene. *J. Biol. Chem.*, **263**, 15335-15341.

FENTON AJ, KEMP BE, HAMMONDS RG, MITCHELHILL K, MOSELEY JM, MARTIN TJ AND NICHOLSON GC. (1991). A potent inhibitor of osteoclastic bone resorption within a highly conserved pentapeptide region of parathyroid hormone-related protein; PTHrP(107-111). *Endocrinol.*, **129**, 3424-3426.

GLATZ JA, HEATH JK, SOUTHBY J, O'KEEFFE LM, KIRIYAMA T, MOSELEY JM, MARTIN TJ AND GILLESPIE MT. (1994). Dexamethasone regulation of parathyroid hormone-related protein (PTHrP) expression in a squamous cancer cell line. *Mol. Cell. Endocrinol.*, **101**, 295-306.

HENDERSON J, SEBAG M, RHIM J, GOLTZMAN D AND KREMER R. (1991). Dysregulation of parathyroid hormone-related peptide expression and secretion in a keratinocyte model of tumor progression. *Cancer Res.*, **51**, 6521-6528.

IKEDA K, WEIR EC, MANGIN M, DANNIES PS, KINDER B, DEFTOS LJ, BROWN EM AND BROADUS AE. (1988). Expression of messenger ribonucleic acids encoding a parathyroid hormone-like peptide in normal human and animal tissues with abnormal expression in human parathyroid adenomas. *Mol. Endocrinol.*, **2**, 1230-1236.

MANGIN M, WEBB AC, DREYER BE, POSILLICO JT, IKEDA K, WEIR EC, STEWART AF, BANDER NH, MILSTONE L, BARTON DE, FRANCKE U AND BROADUS AE. (1988*a*). Identification of a cDNA encoding a parathyroid hormone-related peptide from a human tumor associated with humoral hypercalcemia of malignancy. *Proc. Natl Acad. Sci. USA*, **85**, 597-601.

MANGIN M, IKEDA K, DREYER BE, MILSTONE L AND BROADUS AE. (1988*b*). Two distinct tumor-derived, parathyroid hormone-like peptides result from alternative ribonucleic acid splicing. *Mol. Endocrinol.*, **2**, 1049-1055.

MANGIN M, IKEDA I, DREYER BE AND BROADUS AE. (1989). Isolation and characterization of the human parathyroid hormone-like peptide gene. *Proc. Natl Acad. Sci. USA*, **86**, 2408–2412.

MANGIN M, IKEDA K, DREYER BE AND BROADUS AE. (1990). Identification of an upstream promoter of the human parathyroid hormone-related peptide gene. *Mol. Endocrinol.*, **4**, 851–858.

MARTIN TJ, MOSELEY JM AND GILLESPIE MT. (1991). Parathyroid hormone-related protein: biochemistry and molecular biology. *Crit. Rev. Biochem. Mol. Biol.*, **26**, 377–395.

MOSELEY JM AND GILLESPIE MT. (1992). Parathyroid hormone-related protein. In *Cytokines and Bone Metabolism*, Gowen M (ed.) pp. 325–359. CRC Press: Boca Raton, FL.

RODDA CP, KUBOTA M, HEATH JA, EBELING PR, MOSELEY JM, CARE AD, CAPLE IW AND MARTIN TJ. (1988). Evidence for a novel parathyroid hormone-related protein in fetal lamb parathyroid glands and sheep placenta: comparisons with a similar protein implicated in humoral hypercalcemia of malignancy. *J. Endocrinol.*, **117**, 261–271.

SCHIPANI E, KARGA H, KARAPLIS AC, POTTS Jr JT, KRONENBERG HM, SEGRE GV, ABOU-SAMRA A-B AND JUPPNER H. (1993). Identical complementary deoxyribonucleic acids encode a human renal and bone parathyroid hormone (PTH)/PTH-related peptide receptor. *Endocrinol.*, **132**, 2157–2165.

SOUTHBY J, KISSIN MW, DANKS JA, HAYMAN JA, MOSELEY JM, HENDERSON MA, BENNETT RC AND MARTIN TJ. (1990). Immunohistochemical localization of parathyroid hormone-related protein in human breast cancer. *Cancer Res.*, **50**, 7710–7716.

SOUTHBY J, O'KEEFFE LM, MARTIN TJ AND GILLESPIE MT. (1993). Alternative splicing and promoter usage of the human parathyroid hormone-related protein gene (abstract). *J. Bone Min. Res.*, **8** (suppl. 1) A225.

SUVA LJ, WINSLOW GA, WETTENHALL REH, HAMMONDS RG, MOSELEY JM, DIEFENBACH-JAGGER H, RODDA CP, KEMP BE, RODRIGUEZ H, CHEN EY, HUDSON PJ, MARTIN TJ AND WOOD WI. (1987). A parathyroid hormone-related protein implicated in malignant hypercalcemia: cloning and expression. *Science*, **237**, 893–896.

SUVA LJ, MATHER KA, GILLESPIE MT, WEBB GC, NG KW, WINSLOW GA, WOOD WI, MARTIN TJ AND HUDSON PJ. (1989). Structure of the 5′ flanking region of the gene encoding human parathyroid-hormone-related protein. *Gene*, **77**, 95–105.

THIEDE MA, STREWLER GJ, NISSENSON RA, ROSENBLATT M AND RODAN GA. (1988). Human renal carcinoma expresses two messages encoding a parathyroid hormone-like peptide: evidence for alternative splicing of a single-copy gene. *Proc. Natl Acad. Sci. USA*, **85**, 4605–4609.

TSO JY, SUN X-H, KAO T-H, REECE KS AND WU R. (1985). Isolation and characterization of rat and human glyceraldehyde-3-phosphate dehydrogenase cDNAs: genomic complexity and molecular evolution of the gene. *Nucleic Acids Res.*, **13**, 2485–2502.

URENA P, KONG X-F, ABOU-SAMRA A-B, JUPPNER H, KRONENBERG HM, POTTS Jr JT AND SEGRE GV. (1993). Parathyroid hormone (PTH)/PTH-related peptide receptor messenger ribonucleic acids are widely distributed in rat tissues. *Endocrinology*, **133**, 617–623.

VASAVADA RC, WYSOLMERSKI JJ, BROADUS AE AND PHILBRICK WM. (1993). Identification and characterization of a GC-rich promoter of the human parathyroid hormone-related peptide gene. *Mol. Endocrinol.*, **7**, 273–282.

WINQUIST RJ, BASKIN EP AND VLASUK GP. (1987). Synthetic tumor-derived human hypercalcemic factor exhibits parathyroid hormone-like vasorelaxation in renal arteries. *Biochem. Biophys. Res. Comm.*, **149**, 227–232.

YASUDA T, BANVILLE D, HENDY GN AND GOLTZMAN D. (1989). Characterization of the human parathyroid hormone-like peptide gene. *J. Biol. Chem.*, **264**, 7720–7725.


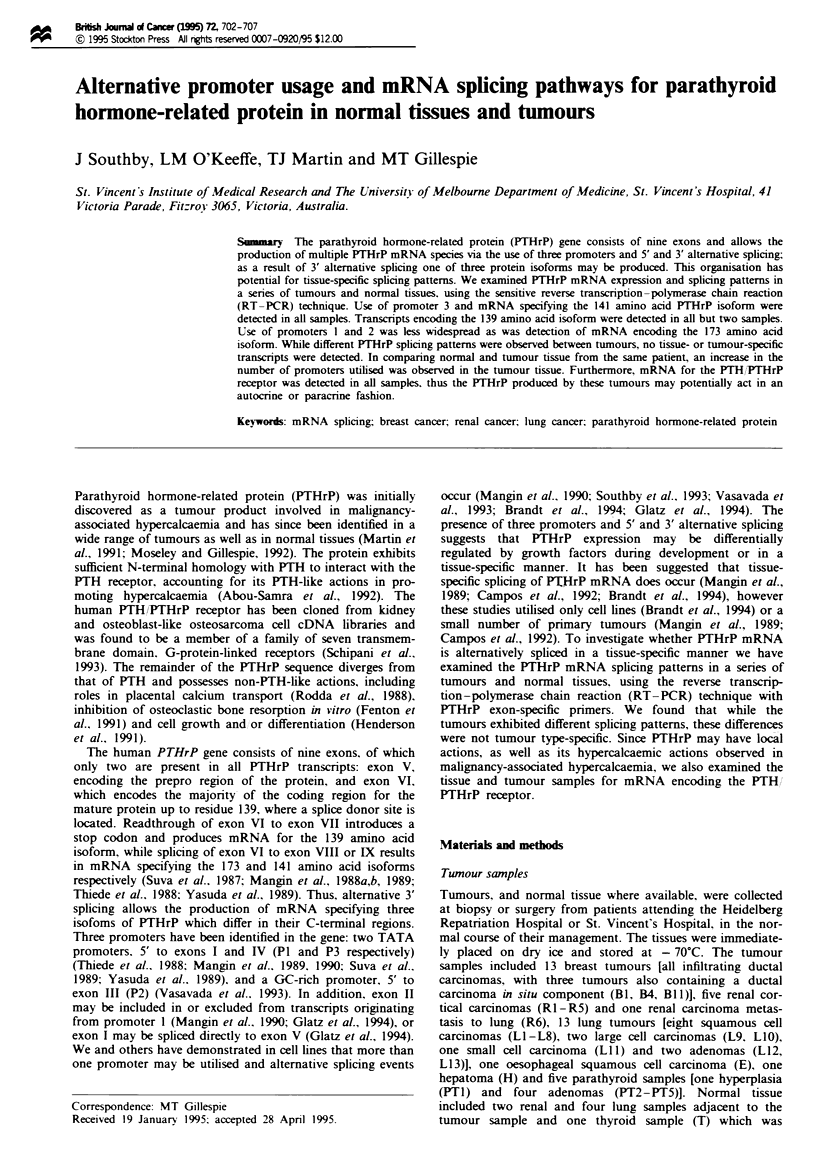

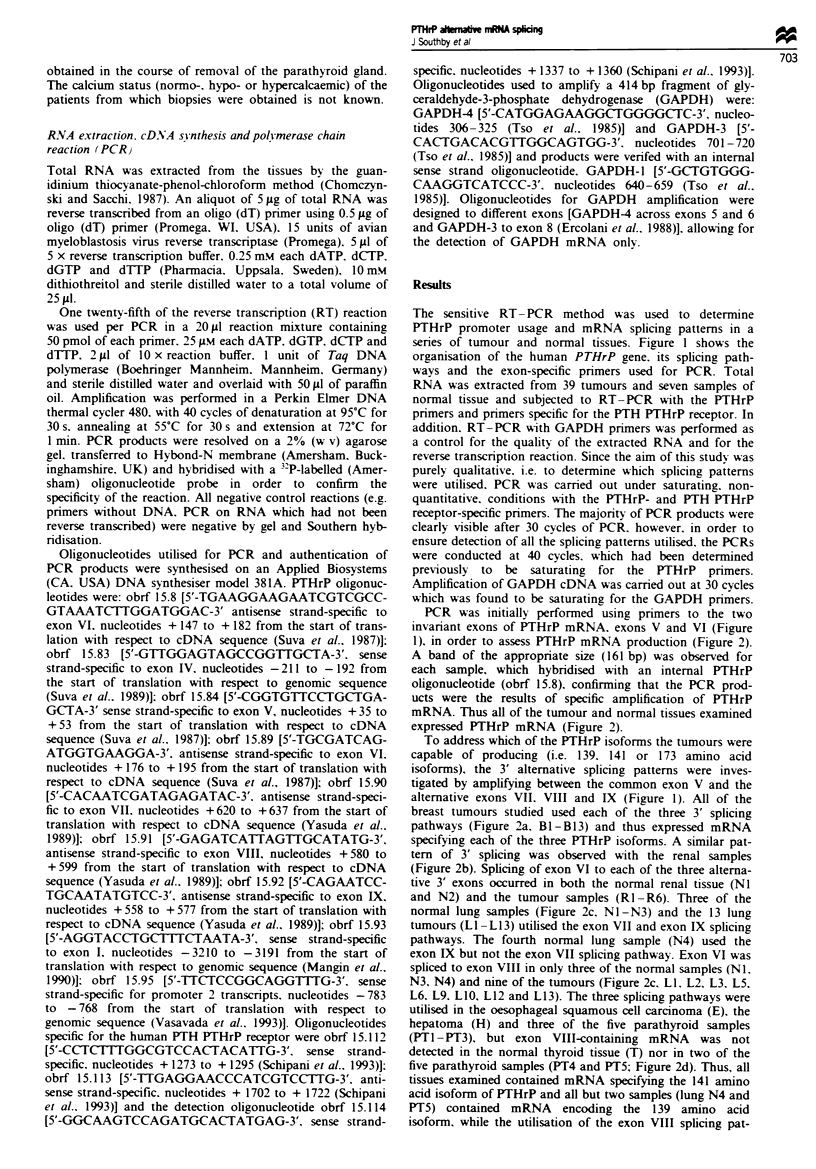

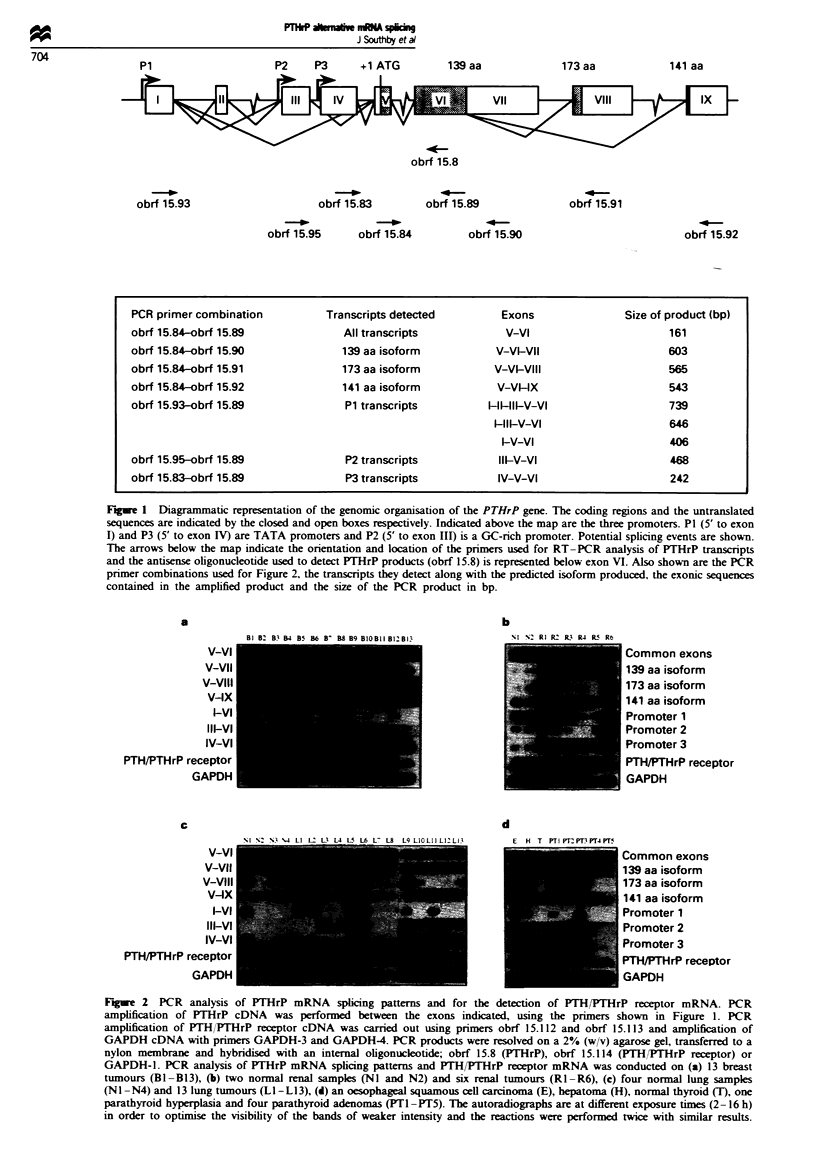

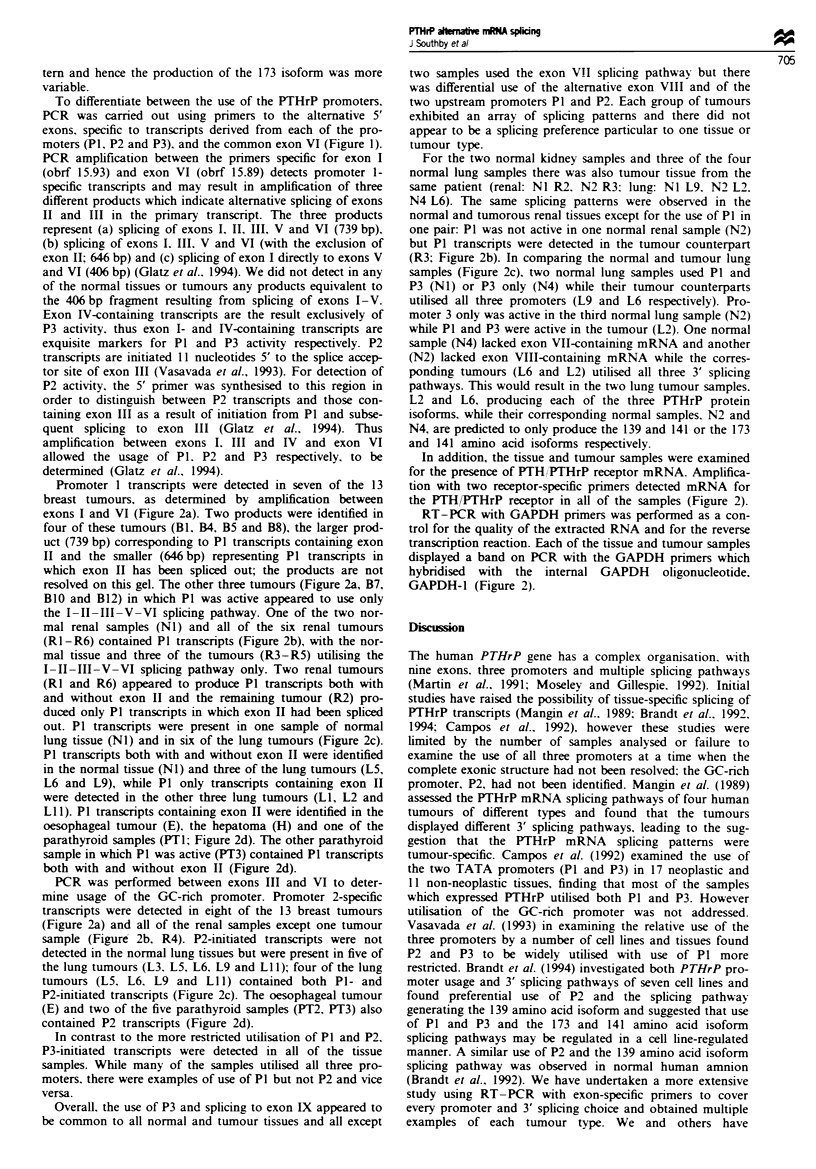

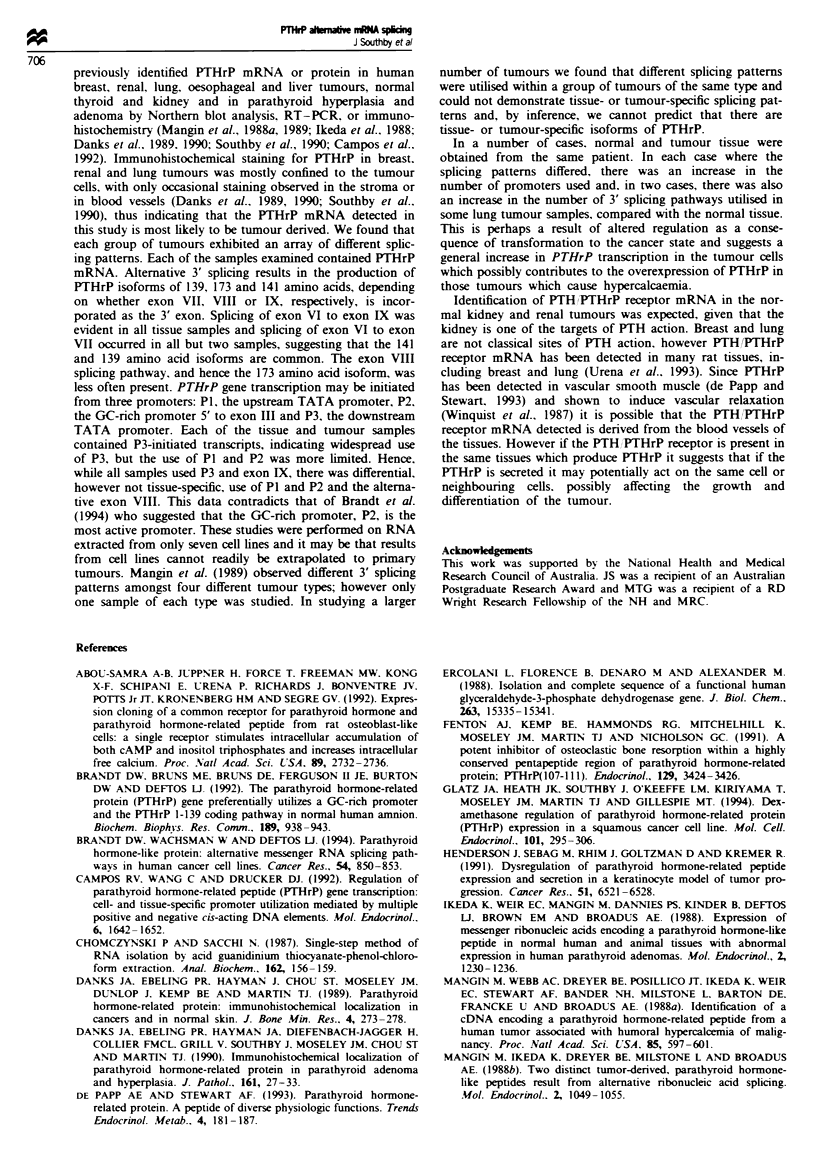

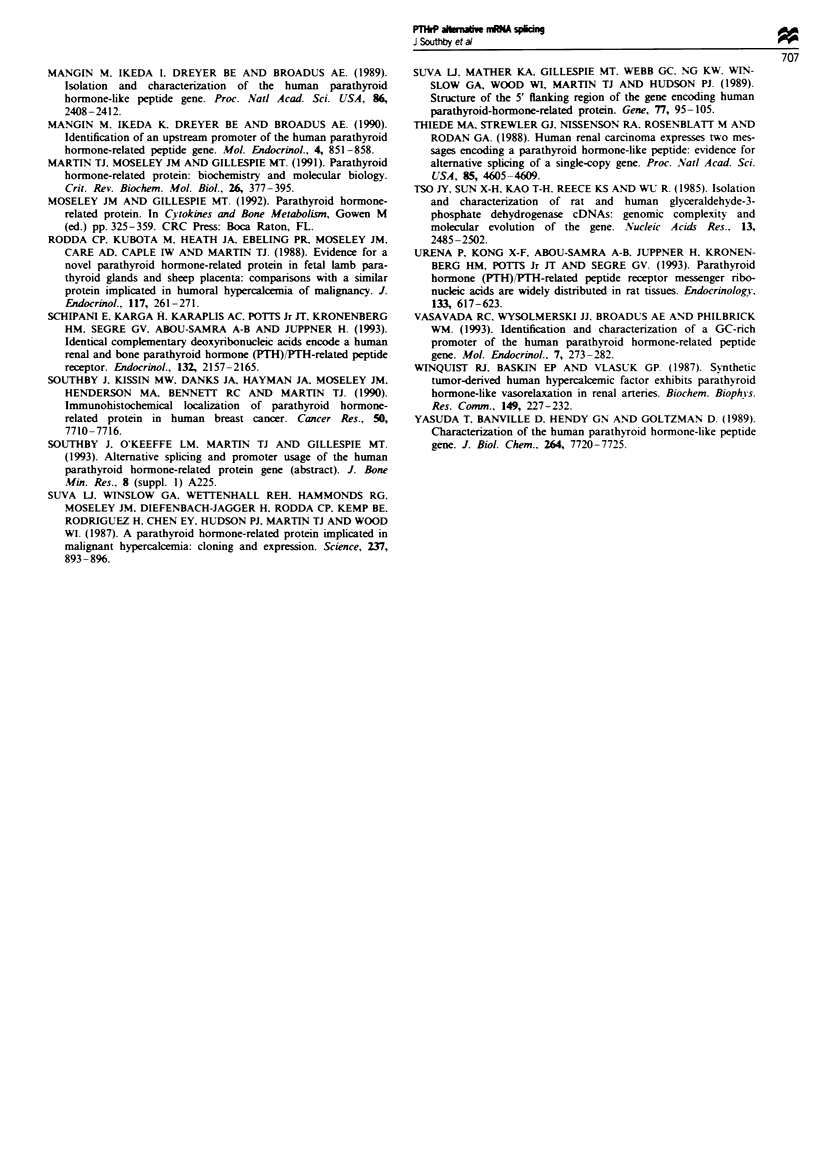


## References

[OCR_00680] Brandt D. W., Bruns M. E., Bruns D. E., Ferguson J. E., Burton D. W., Deftos L. J. (1992). The parathyroid hormone-related protein (PTHrP) gene preferentially utilizes a GC-rich promoter and the PTHrP 1-139 coding pathway in normal human amnion.. Biochem Biophys Res Commun.

[OCR_00686] Brandt D. W., Wachsman W., Deftos L. J. (1994). Parathyroid hormone-like protein: alternative messenger RNA splicing pathways in human cancer cell lines.. Cancer Res.

[OCR_00691] Campos R. V., Wang C., Drucker D. J. (1992). Regulation of parathyroid hormone-related peptide (PTHrP) gene transcription: cell- and tissue-specific promoter utilization mediated by multiple positive and negative cis-acting DNA elements.. Mol Endocrinol.

[OCR_00698] Chomczynski P., Sacchi N. (1987). Single-step method of RNA isolation by acid guanidinium thiocyanate-phenol-chloroform extraction.. Anal Biochem.

[OCR_00709] Danks J. A., Ebeling P. R., Hayman J. A., Diefenbach-Jagger H., Collier F. M., Grill V., Southby J., Moseley J. M., Chou S. T., Martin T. J. (1990). Immunohistochemical localization of parathyroid hormone-related protein in parathyroid adenoma and hyperplasia.. J Pathol.

[OCR_00703] Danks J. A., Ebeling P. R., Hayman J., Chou S. T., Moseley J. M., Dunlop J., Kemp B. E., Martin T. J. (1989). Parathyroid hormone-related protein: immunohistochemical localization in cancers and in normal skin.. J Bone Miner Res.

[OCR_00721] Ercolani L., Florence B., Denaro M., Alexander M. (1988). Isolation and complete sequence of a functional human glyceraldehyde-3-phosphate dehydrogenase gene.. J Biol Chem.

[OCR_00725] Fenton A. J., Kemp B. E., Hammonds R. G., Mitchelhill K., Moseley J. M., Martin T. J., Nicholson G. C. (1991). A potent inhibitor of osteoclastic bone resorption within a highly conserved pentapeptide region of parathyroid hormone-related protein; PTHrP[107-111].. Endocrinology.

[OCR_00735] Glatz J. A., Heath J. K., Southby J., O'Keeffe L. M., Kiriyama T., Moseley J. M., Martin T. J., Gillespie M. T. (1994). Dexamethasone regulation of parathyroid hormone-related protein (PTHrP) expression in a squamous cancer cell line.. Mol Cell Endocrinol.

[OCR_00739] Henderson J., Sebag M., Rhim J., Goltzman D., Kremer R. (1991). Dysregulation of parathyroid hormone-like peptide expression and secretion in a keratinocyte model of tumor progression.. Cancer Res.

[OCR_00745] Ikeda K., Weir E. C., Mangin M., Dannies P. S., Kinder B., Deftos L. J., Brown E. M., Broadus A. E. (1988). Expression of messenger ribonucleic acids encoding a parathyroid hormone-like peptide in normal human and animal tissues with abnormal expression in human parathyroid adenomas.. Mol Endocrinol.

[OCR_00780] Mangin M., Ikeda K., Dreyer B. E., Broadus A. E. (1990). Identification of an up-stream promoter of the human parathyroid hormone-related peptide gene.. Mol Endocrinol.

[OCR_00772] Mangin M., Ikeda K., Dreyer B. E., Broadus A. E. (1989). Isolation and characterization of the human parathyroid hormone-like peptide gene.. Proc Natl Acad Sci U S A.

[OCR_00764] Mangin M., Ikeda K., Dreyer B. E., Milstone L., Broadus A. E. (1988). Two distinct tumor-derived, parathyroid hormone-like peptides result from alternative ribonucleic acid splicing.. Mol Endocrinol.

[OCR_00756] Mangin M., Webb A. C., Dreyer B. E., Posillico J. T., Ikeda K., Weir E. C., Stewart A. F., Bander N. H., Milstone L., Barton D. E. (1988). Identification of a cDNA encoding a parathyroid hormone-like peptide from a human tumor associated with humoral hypercalcemia of malignancy.. Proc Natl Acad Sci U S A.

[OCR_00785] Martin T. J., Moseley J. M., Gillespie M. T. (1991). Parathyroid hormone-related protein: biochemistry and molecular biology.. Crit Rev Biochem Mol Biol.

[OCR_00796] Rodda C. P., Kubota M., Heath J. A., Ebeling P. R., Moseley J. M., Care A. D., Caple I. W., Martin T. J. (1988). Evidence for a novel parathyroid hormone-related protein in fetal lamb parathyroid glands and sheep placenta: comparisons with a similar protein implicated in humoral hypercalcaemia of malignancy.. J Endocrinol.

[OCR_00801] Schipani E., Karga H., Karaplis A. C., Potts J. T., Kronenberg H. M., Segre G. V., Abou-Samra A. B., Jüppner H. (1993). Identical complementary deoxyribonucleic acids encode a human renal and bone parathyroid hormone (PTH)/PTH-related peptide receptor.. Endocrinology.

[OCR_00811] Southby J., Kissin M. W., Danks J. A., Hayman J. A., Moseley J. M., Henderson M. A., Bennett R. C., Martin T. J. (1990). Immunohistochemical localization of parathyroid hormone-related protein in human breast cancer.. Cancer Res.

[OCR_00832] Suva L. J., Mather K. A., Gillespie M. T., Webb G. C., Ng K. W., Winslow G. A., Wood W. I., Martin T. J., Hudson P. J. (1989). Structure of the 5' flanking region of the gene encoding human parathyroid-hormone-related protein (PTHrP).. Gene.

[OCR_00821] Suva L. J., Winslow G. A., Wettenhall R. E., Hammonds R. G., Moseley J. M., Diefenbach-Jagger H., Rodda C. P., Kemp B. E., Rodriguez H., Chen E. Y. (1987). A parathyroid hormone-related protein implicated in malignant hypercalcemia: cloning and expression.. Science.

[OCR_00835] Thiede M. A., Strewler G. J., Nissenson R. A., Rosenblatt M., Rodan G. A. (1988). Human renal carcinoma expresses two messages encoding a parathyroid hormone-like peptide: evidence for the alternative splicing of a single-copy gene.. Proc Natl Acad Sci U S A.

[OCR_00842] Tso J. Y., Sun X. H., Kao T. H., Reece K. S., Wu R. (1985). Isolation and characterization of rat and human glyceraldehyde-3-phosphate dehydrogenase cDNAs: genomic complexity and molecular evolution of the gene.. Nucleic Acids Res.

[OCR_00852] Ureña P., Kong X. F., Abou-Samra A. B., Jüppner H., Kronenberg H. M., Potts J. T., Segre G. V. (1993). Parathyroid hormone (PTH)/PTH-related peptide receptor messenger ribonucleic acids are widely distributed in rat tissues.. Endocrinology.

[OCR_00858] Vasavada R. C., Wysolmerski J. J., Broadus A. E., Philbrick W. M. (1993). Identification and characterization of a GC-rich promoter of the human parathyroid hormone-related peptide gene.. Mol Endocrinol.

[OCR_00862] Winquist R. J., Baskin E. P., Vlasuk G. P. (1987). Synthetic tumor-derived human hypercalcemic factor exhibits parathyroid hormone-like vasorelaxation in renal arteries.. Biochem Biophys Res Commun.

[OCR_00868] Yasuda T., Banville D., Hendy G. N., Goltzman D. (1989). Characterization of the human parathyroid hormone-like peptide gene. Functional and evolutionary aspects.. J Biol Chem.

